# Game Plan—a Brief Web-Based Intervention to Improve Uptake and Use of HIV Pre-exposure Prophylaxis (PrEP) and Reduce Alcohol Use Among Gay and Bisexual Men: Content Analysis

**DOI:** 10.2196/30408

**Published:** 2022-01-05

**Authors:** Tyler B Wray, Philip A Chan, John P Guigayoma, Christopher W Kahler

**Affiliations:** 1 Department of Behavioral and Social Sciences School of Public Health Brown University Providence, RI United States; 2 Department of Medicine Warren Alpert Medical School Brown University Providence, RI United States

**Keywords:** HIV, pre-exposure prophylaxis, alcohol use, mHealth, eHealth, intervention, mobile phone

## Abstract

**Background:**

HIV pre-exposure prophylaxis (PrEP) has considerable potential for reducing incidence among high-risk groups, such as gay, bisexual, and other men who have sex with men (GBM). However, PrEP’s effectiveness is closely linked with consistent use, and a variety of individual-level barriers, including alcohol use, could impede optimal uptake and use. Web-based interventions can encourage medication adherence, HIV prevention behaviors, and responsible drinking and may help support PrEP care, particularly in resource-limited settings.

**Objective:**

We previously developed a web application called Game Plan that was designed to encourage heavy drinking GBM to use HIV prevention methods and reduce their alcohol use and was inspired by brief motivational interventions. This paper aims to describe the web-based content we designed for integration into Game Plan to help encourage PrEP uptake and consistent use among GBM. In this paper, we also aim to describe this content and its rationale.

**Methods:**

Similar to the original site, these components were developed iteratively, guided by a thorough user-centered design process involving consultation with subject-matter experts, usability interviews and surveys, and user experience surveys.

**Results:**

In addition to Game Plan’s pre-existing content, the additional PrEP components provide specific, personal, and digestible feedback to users about their level of risk for HIV without PrEP and illustrate how much consistent PrEP use could reduce it; personal feedback about their risk for common sexually transmitted infections to address low-risk perceptions; content challenging common beliefs and misconceptions about PrEP to reduce stigma; content confronting familiar PrEP and alcohol beliefs; and a change planning module that allows users to select specific goals for starting and strategies for consistent PrEP use. Users can opt into a weekly 2-way SMS text messaging program that provides similar feedback over a 12-week period after using Game Plan and follows up on the goals they set.

**Conclusions:**

Research preliminarily testing the efficacy of these components in improving PrEP outcomes, including uptake, adherence, sexually transmitted infection rates, and alcohol use, is currently ongoing. If supported, these components could provide a scalable tool that can be used in resource-limited settings in which face-to-face intervention is difficult.

## Introduction

### Background

Although the rates of new HIV infections have declined among most groups in the United States in the recent years, incidence remains consistently high among gay, bisexual, and other men who have sex with men (GBM) [1,2]. Daily oral HIV pre-exposure prophylaxis (PrEP) is an extremely effective HIV prevention option and has the potential to reduce incidence in this group [3,4]. Although the US Food and Drug Administration approved PrEP for adults in 2012 [5], PrEP uptake remains lower than that needed to achieve stable declines in new infections [6,7]. PrEP’s efficacy is also closely linked to adherence [8], and although recent demonstration projects and real-world studies have shown high adherence among many GBM [9,10], discontinuation is also common [11]. Young GBM may start PrEP at lower rates, show suboptimal adherence, and have higher rates of discontinuation [10,12]. In some demonstration projects focusing on younger GBM, only 34% of the participants had protective levels of PrEP after 48 weeks [10], far less than the average of about 2 years in which many GBM are at the highest risk for HIV [13]. The rates of other sexually transmitted infections (STIs) are also particularly high among GBM who use PrEP [14]. Efficient, cost-effective interventions are needed that can encourage broader PrEP uptake and consistent use and help reduce STI rates among PrEP users.

Factors such as access and cost have consistently been among the most important barriers to PrEP use among GBM [15,16], but improved coverage among health insurers and national programs providing free PrEP for the uninsured could significantly reduce these barriers [17,18]. However, even with equal access, a number of other important barriers limit PrEP use in GBM. Previous research has shown that low perceived risk for HIV [19,20], low social norms around PrEP (particularly among racial or ethnic minority GBM) [21,22], persistent PrEP stigma [23], and low uptake and adherence self-efficacy [24,25] are among the most important factors determining PrEP use. Unhealthy alcohol use could also make success on PrEP difficult. Alcohol use is a major risk factor for HIV acquisition [26], primarily because intoxication can interfere with condom use during sex in GBM [27,28]. Given this link, it is important to encourage PrEP uptake among GBM who drink heavily. Some evidence suggests that heavy drinking GBM may be less willing to use PrEP and may need increased guidance and support to start PrEP [29]. Low condom use among GBM on PrEP in general also contributes to high rates of STIs [30,31], and heavy drinking GBM could be at even higher risk for STIs when on PrEP [32,33]. For these reasons, interventions to improve PrEP use should provide STI-risk reduction counseling for all PrEP users, and this may be especially helpful for heavy drinking GBM. Finally, although there is little evidence to date that alcohol use interferes with PrEP adherence [34,35], interactive toxicity beliefs are common [29], and lifestyle disruption because of heavy drinking may also confer some risk for suboptimal adherence [34,36].

In clinical settings, interventions to encourage PrEP uptake and optimize PrEP outcomes among those taking PrEP are not consistently available. Optimizing patient outcomes on PrEP typically requires a high level of ongoing follow-up and support in the form of encouraging uptake, supporting adherence, providing ongoing monitoring, and preventing discontinuation among those who would still benefit from PrEP [37]. Therefore, national guidelines and previous research have underscored the value of providing support or counseling interventions for PrEP patients alongside typical PrEP monitoring and care [38,39]. However, routinely providing this degree of support to PrEP patients in practice is a concern [40], even among dedicated PrEP clinics. Encouraging nonspecialty providers, such as family medicine and primary care clinicians, to provide PrEP has been a key strategy for expanding PrEP access [41,42], but providers in these settings may feel even less equipped to provide an intensive level of ongoing support that is often recommended for optimal PrEP care [40], which may be pronounced in lower resource settings.

A variety of individual-level interventions for improving PrEP outcomes have been designed to date, but nearly all have yet to be rigorously tested [43,44]. Most of these interventions also rely on some form of face-to-face counseling and often require highly trained counselors to meet with patients for several hours [45,46]. These characteristics are resource-intensive and may impede implementation, particularly in low-resource PrEP clinics and nonspecialty settings. A brief intervention that uses evidence-based techniques to address key factors involved in PrEP uptake, adherence, and persistence and is relatively inexpensive and highly feasible across a variety of settings may help provide patients with basic information and support that help them to be more successful on PrEP.

Meta-analyses and systematic reviews have consistently shown that web-based interventions can encourage HIV prevention behaviors [47,48] and could overcome many of the implementation issues of current PrEP support interventions. Web-based interventions are also easy to disseminate and cheaper than individual counseling, making their widespread implementation much more feasible than other approaches [49,50]. They can also standardize content, which is an important benefit given that other interventions delivered by counselors struggle with fidelity and drift as dissemination increases [51]. Web-based interventions are also easier for individual users to access than smartphone apps because they do not require downloading an app and can be used from a variety of devices. Finally, they may also be particularly well-suited to reach young GBM, a key risk group given high rates of HIV and low PrEP uptake and use [1,10,12]. Given these strengths, we designed and built the components of a web application to help improve PrEP outcomes in GBM.

### Objective

Specifically, our goal was to design simple, short features that could help (1) encourage GBM who are not already on PrEP to start using PrEP, (2) urge those already on PrEP to take it consistently for as long as they are at risk, and (3) promote condom use and the use of other forms of prevention to reduce their risk of STIs. Given the conceptual overlap in these goals with Game Plan, a previous web application we built to help GBM reduce their risk for HIV [52], we incorporated the features we designed for PrEP-related goals into the broader Game Plan web app. This paper describes the features we designed to help improve PrEP outcomes and their theoretical and evidence-based underpinnings.

## Methods

### Background and Overall Design

Game Plan is a web application that was initially developed primarily to help HIV-negative GBM who are *not* on PrEP to reflect on their choices about sex and other potential risks associated with HIV acquisition such as alcohol use and, if interested, consider ways of changing these behaviors to be safer [53]. It is a self-guided application that provides both static and interactive content. It was explicitly designed to be brief (approximately 20-30 minutes, on average) so that it is feasible for use in clinics (eg, in waiting rooms) or at home and to more closely resemble how many people access and use health information on the web: during a few brief visits [54,55]. In our formative work, users spent 20 to 40 minutes interacting with the application in a single visit [53,56]. The site was originally developed using a thorough user-centered design research process [57] conducted among GBM across the United States but concentrated in the northeastern United States. It primarily uses a wizard navigation pattern in which users are guided through a series of sections and activities with progressive disclosure rather than the typical explorative hierarchy of pages. In a small pilot study, the site showed promising effects on both reducing HIV-risk behavior and alcohol use among high-risk, heavy drinking GBM [58]. A full efficacy trial of Game Plan’s effects on sexual risk behavior and alcohol use among GBM who are *not* on PrEP is currently ongoing.

Although the initial version of Game Plan suggested that users who were interested in reducing their HIV risk consider PrEP, it included only limited PrEP content. This study focused on designing, developing, and testing additional components that were more explicitly intended to encourage PrEP uptake among GBM at high risk but not currently on PrEP and to encourage consistent PrEP use among those already taking PrEP. Given the notably high burden of STIs among GBM on PrEP [59], we also aim to incorporate content intended to encourage the use of condoms and other prevention strategies among those currently taking PrEP. We also incorporated several overall design and feature updates to all Game Plan content.

### Theoretical Foundation and Design Research

The content of Game Plan is generally informed by the Information-Motivation-Behavior model [60,61] and adopts the basic framework of brief motivational interventions [62] and the *spirit* of motivational interviewing (MI) [63]. It is primarily focused on helping users who are not on PrEP move from the precontemplation or contemplation stages to the preparation and action stages of change [64]. For those already on PrEP, it is focused on reinforcing their commitment to change during the maintenance stage. However, it is not intended to provide exhaustive or continuous support over time for those in the maintenance stage. We developed the PrEP-specific content that is the focus of this paper to align with these theories and approaches.

To guide the development of these components, we consulted with other PrEP experts to identify the most pressing goals for PrEP care to help prioritize content. Then, we reviewed the available literature to explore key determinants of these goals and potential behavior change techniques with promise in addressing each one. After outlining the basic content, we worked with a professional design team to create a prototype version of the fully redesigned site that incorporated the new PrEP content. We then conducted a thorough usability interview and surveys with 10 heavy drinking GBM who were currently on PrEP and reported taking <80% (24/30 days) of their daily doses of PrEP in the last month. In these interviews, trained interviewers provided participants with a link to the prototype site and asked them to click through each section and *think aloud* as they did so, explicitly encouraging critical feedback [65]. After reviewing each interview and incorporating changes based on the feedback provided, we conducted a user experience survey [66] with 40 additional heavy drinking GBM who were currently on PrEP. These participants completed a baseline survey, received a link to the prototype site, and then completed a follow-up survey to assess their perceptions of the site. The results of these studies have been published elsewhere [56]. In this paper, we describe each component and its theoretical or empirical basis.

## Results

### Flow, Content, and Onboarding

**Figure 1:**
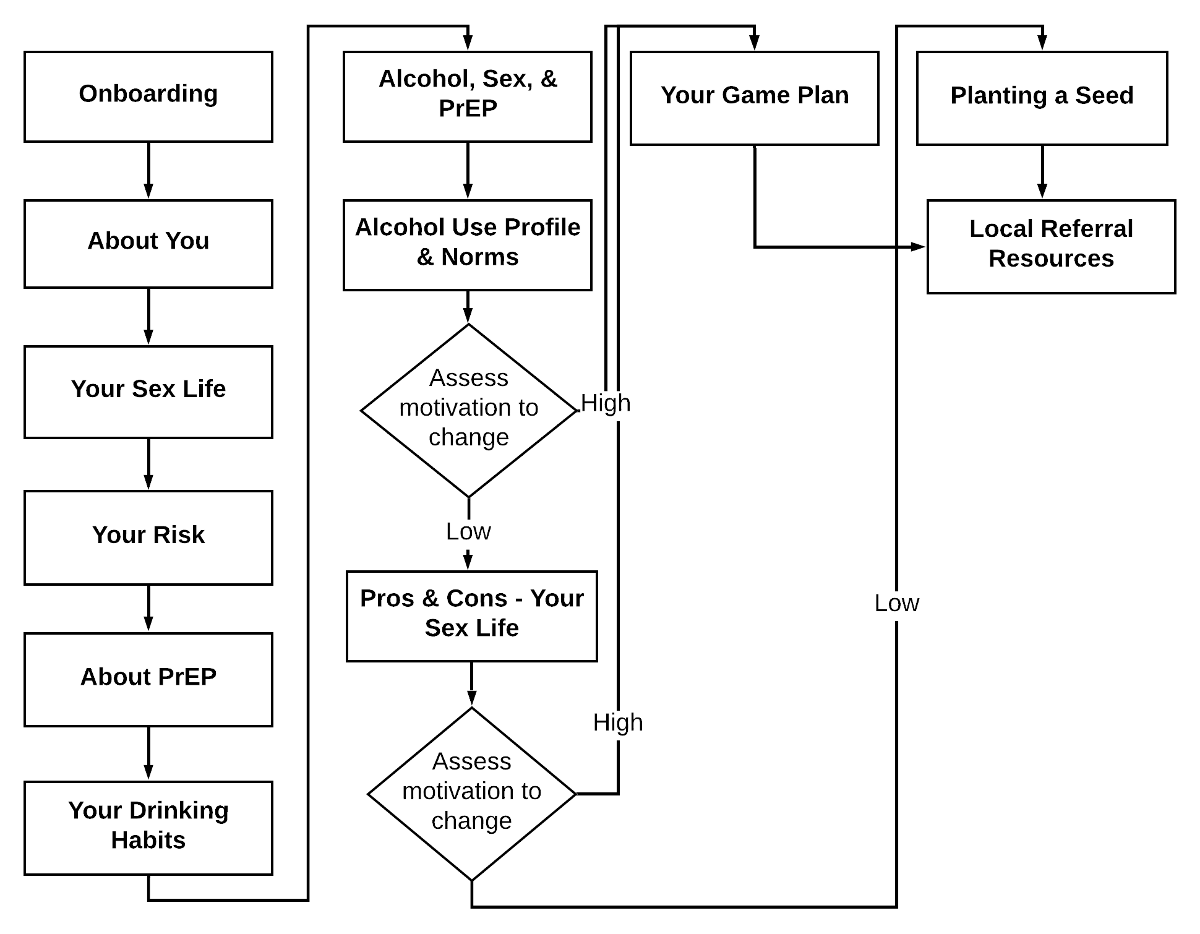
Game Plan web application flow for users reporting the use of pre-exposure prophylaxis. PrEP: pre-exposure prophylaxis.

The redesigned Game Plan site with PrEP-specific content generally followed a similar flow to the original Game Plan, which was intended to align approximately with two phases common in MI: (1) content eliciting intrinsic motivation to change and when and if sufficient motivation exists and (2) content intended to help translate that motivation into specific change plans. The overall sequence first discusses HIV and STI topics, followed by alcohol use. Some new PrEP-specific content is presented conditionally based on whether users reported currently using PrEP or not in the About You section, whereas other content is presented to all users because it may benefit PrEP uptake and PrEP use alike (see Table 1 for a brief description of all components). The flow of all the components is presented in Figure 1.

The following sections describe each section of Game Plan and its content in the order in which it is presented to the users and then cite the theoretical and empirical literature that informed content decisions and design.

**Table 1:** Techniques used in each Game Plan section and theoretical constructs or mechanisms they pursue.

Component	Techniques and goals	Theory constructs addressed
Onboarding	Set “tone” Express empathy	—^a^
About you	Assess fit for user GBM^b^ Assess tailoring variables (age and PrEP^c^use) Assess user’s values	Tailoring Grounding guidance in user’s values
Your sex life	Efficiently assess the number of past-year unknown-status partners and number of CAS^d^ events as top and bottom	Tailoring
Your risk	Affirm decision to use PrEP Show potential HIV risk over 1 and 5 years based on past-year behavior without PrEP Show how much calculated HIV risk percentages would be reduced with consistent PrEP use Compare past-year HIV risk to all men and gay or bisexual men Show potential risk for chlamydia and gonorrhea in the past year with PrEP use Compare past-year number of partners and CAS with other GBM in age group	Motivation (risk perceptions) Motivation (PrEP use) Motivation (social norms)
About PrEP	Challenge common misconceptions and stigma about PrEP Providing information and instrumental support	Information or PrEP knowledge Challenging PrEP stigma
Your drinking habits	Assess users’ frequency of alcohol use over the past month Assess the quantity users drank during each occasion over the past month Assess regret or remorse after drinking and blackouts	Tailoring Motivation (anticipated regret)
Alcohol, sex, and PrEP	Challenge common beliefs about alcohol facilitating sex based on specific motivations selected by users Provide information about alcohol’s potential effects on PrEP adherence Challenge PrEP-alcohol toxicity beliefs	Information
Alcohol use profile and norms	Provide feedback about level of risk associated with current level of alcohol use Compare past-month alcohol use with other GBM in age group Present HIV and STI^e^ risk profile information again to refresh	Motivation (risk perceptions) Motivation (social norms)
Pros and cons exercise	Help users weigh pros and cons of current choices about sex Provide feedback about how these pros and cons stack up Prompt reflection on how these pros and cons align with identified values	Motivation (develop discrepancy)
Your Game Plan	Explore menu of options for ensuring PrEP adherence, reducing STI risk, and reducing alcohol use Identify important reasons for making selected changes Choose specific steps for working toward goal Provide referrals for prevention services (STI testing, PrEP, etc)	Self-efficacy Information (PrEP adherence strategies, instrumental support, and other prevention strategies) Motivation (commitment to change plan)
Planting a seed	Prompt users to consider what would need to happen to consider change Encourage revisiting the site if things change	Self-efficacy
Local resources	Provide information about prevention services (STI testing and PrEP) and other health care services (medical, mental health, and drug or alcohol treatment) tailored to their area Display PrEP or HIV service locator widgets	Self-efficacy Information (instrumental support)

^a^No theory-based constructs or mechanisms are addressed for this content because it is only intended to help orient the user to the program.

^b^GBM: gay, bisexual, and other men who have sex with men.

^c^PrEP: pre-exposure prophylaxis.

^d^CAS: condomless anal sex.

^e^STI: sexually transmitted infection.

### Onboarding

After the splash screen, users first see a single onboarding screen that explains the purpose of Game Plan and is intended to express empathy and convey other aspects of the MI *spirit*: that the site is nonjudgmental, collaborative, and respects users’ autonomy [63]. Next, the *About You* section requests basic information about the user to tailor the content presented throughout the site, including age, gender, sex at birth, sexual orientation, PrEP use, and location [67]. No identifying information is collected, as the site was specifically designed to be anonymous unless the user volunteers to provide their contact information at the end for further follow-up. The *About You* section also asks users to consider a list of values (eg, adventure—having variety, excitement, justice—being fair and accurate, mastery—achievement, challenge, and growth) and asks users to choose up to 4 that they aspire to in their own lives. The goal of this exercise is to encourage users to keep these values in mind as they consider change and whether their current choices align with values that are important to them. Therefore, the user’s selected values are summarized for them on the change goals page.

### Your Sex Life

This section was designed to assess variables needed to approximate users’ level of risk for HIV and other common STIs (chlamydia, gonorrhea, syphilis, etc) in as few questions as possible. Because condomless anal sex (CAS) with partners of uncertain HIV or STI status confers at least some risk, these questions are focused primarily on helping users report the number of *times* they had CAS as a top and bottom with these partners. These responses are then used to estimate HIV risk over a year and over 5 years, with and without PrEP, and other STI risk in the following sections.

### Your Risk Profile

Users are then presented with a *risk profile* that provides personal, easy-to-digest estimates of their risk based on the data collected in the previous section. As in the original Game Plan, the profile starts by reporting estimates of their risk for HIV over the next year and 5 years if their sex life remains the same, using data from past research [68]. This estimate is calculated using data that the users have entered about the number of condomless, insertive and receptive anal sex events with unknown–HIV-status partners, together with national estimates of HIV prevalence in GBM and average per-act transmission risks. The goal of this step was to correct any misperceptions of personal risk by providing a credible, personally relevant, and digestible sense of their risk level. Several studies have shown that underestimating one’s risk is a key barrier to PrEP uptake in GBM [19,69]. Users are then shown what their HIV risk would be if they used PrEP every day, using estimates drawn from effectiveness studies [70,71]. By presenting credible estimates of a user’s personal risk with and without PrEP side by side, our goal was to highlight the significant impact that PrEP could have on their personal risk. One of the key principles of MI suggests that one way to enhance users’ intrinsic motivation for change is to draw their attention to discrepancies [63], for example, between an individual’s current behavior (not taking PrEP) and desired future states (remaining HIV-negative). Some research in addiction suggests that individuals who experience stronger actual-ideal discrepancies such as these after the intervention show greater change in alcohol or drug use [72,73]. Thus, we hope that highlighting this discrepancy between current risk and much lower potential risk with PrEP might increase users’ intrinsic motivation to use PrEP. Finally, using national survey data [74], the risk profile also shows how the users’ total number of sex partners and number of CAS events compare with other GBM in the same age group. This step was intended to illustrate the extent to which the user’s current risk and behavior deviates from the norms of valued reference groups [73]. Correcting inaccurate perceptions about the social norms of given behaviors may similarly enhance motivation to change by increasing the discrepancy that users experience between current behavior and a desired outcome (avoiding risk behavior that is too extreme compared with that of others) [75,76].

**Figure 2:**
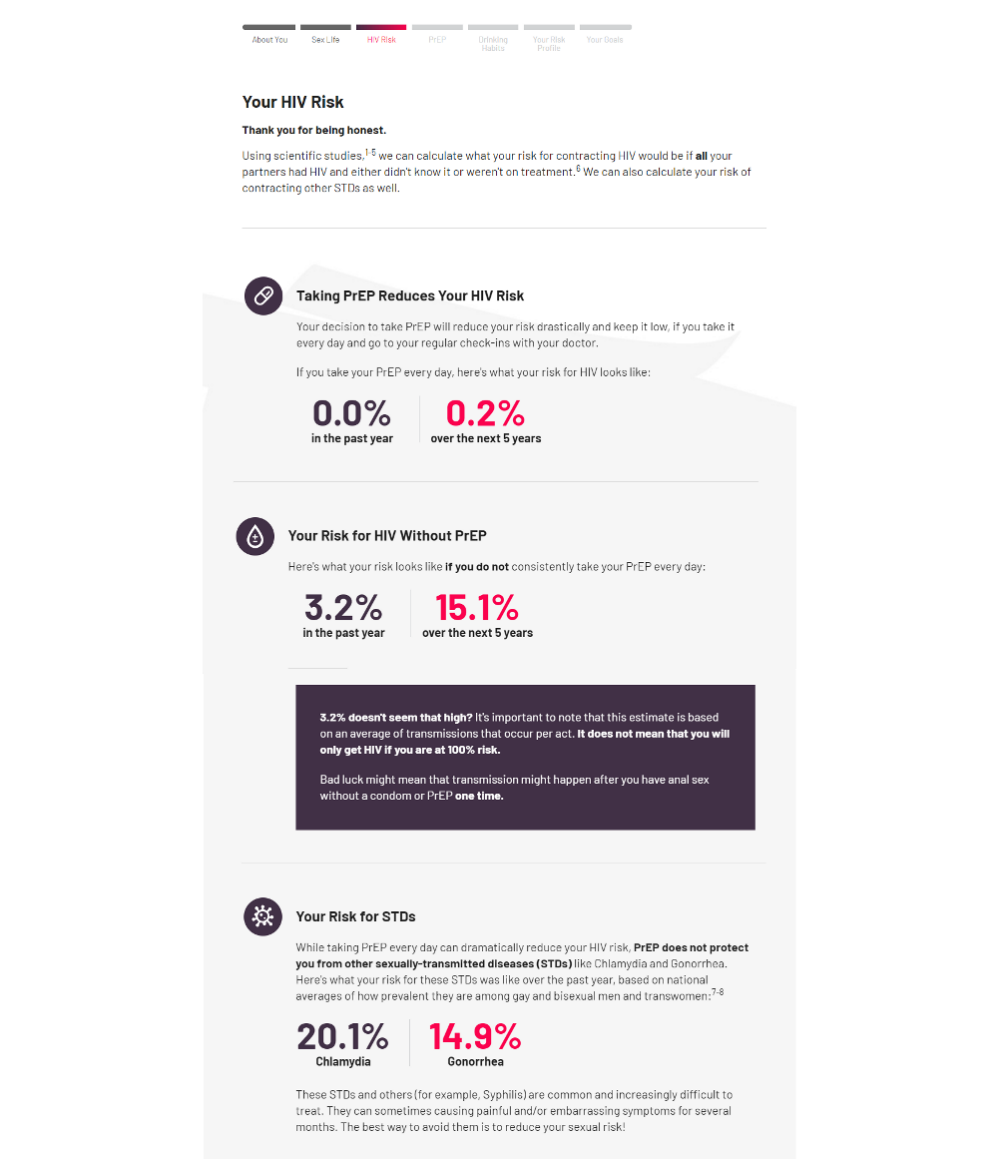
Game Plan—HIV risk profile. PrEP: pre-exposure prophylaxis.

For users already using PrEP, the risk profile starts by affirming their decision to use PrEP. Then, similar to PrEP nonusers, their risk profile displays estimates of HIV risk over a year and 5 years given current behavior if they *did not* use PrEP, followed by the same estimates adjusted for taking PrEP every day. Highlighting this difference in those already using PrEP could similarly boost these users’ motivation to continue using PrEP for as long as they are at risk and to continue taking it daily during that time. However, below the risk profile for current PrEP users, the profile emphasizes the importance of continuing to use prevention methods (eg, condoms) while on PrEP because of the continued risk for other STIs and provides estimates of likely past-year personal risk for chlamydia and gonorrhea using data from past research [77,78]. As with HIV, the goal of this step was to increase motivation to reduce their risk for other STIs by correcting inaccurate risk perceptions. Similar to PrEP nonusers, the risk profile for PrEP users also provides normative comparisons for past-year sexual behavior compared with that of other GBM in their age group to increase motivation to use additional prevention methods. Afterward, the site assesses users’ reactions to this information as well as their motivation to change to reduce their risk (Figure 2).

At least one large study has tested the effects of a similar technique using a calculator to give GBM feedback about their risk for HIV based on their recent behavior on PrEP uptake among GBM who were not on PrEP [69]. This risk calculator used participants’ responses to 16 questions relevant to risk (eg, CAS over the last 30 days, recent STIs, and drug use) to provide 2 risk scores to participants, which were presented both via iPad and verbally. Results suggested that GBM who were provided with these scores were not more likely to start PrEP after 8 weeks than those who were not, despite a substantial number of participants underestimating their risk at baseline. Although these findings could suggest that providing GBM with more objective information about their risk does not lead to greater PrEP uptake in general, the extent to which the specific scores used in this study increased the accuracy of participants’ misperceptions is not clear. It is also possible that the specific type of feedback given, which involved classifying participants into broad low-, medium-, and high-risk categories, may not be as powerful as providing more specific values. Providing feedback such as this could also be more effective when users are also directly linked to options for actions they can take to reduce their risk, and additional content is also dedicated to easing key barriers (eg, PrEP stigma). Although there could be some concern that providing such specific estimates may inadvertently convey certainty about users’ risk level, text boxes and footnotes on this page specifically highlight that their risk level is an *estimate* that is calculated using *average* per-act risk and that their true risk also depends on several other unassessed factors (eg, whether either partner had another STI and how recently the HIV-positive partner was infected). If this basic technique proves to be helpful, we intend to revisit it in future versions to tailor this feedback based on other important factors (eg, HIV prevalence in the user’s age group and other demographics) and consider how to present this information even more carefully.

### About PrEP

The goal of the About PrEP section is to provide information that primarily addresses common misconceptions about PrEP and challenges beliefs that contribute to stigmas about PrEP use. We included this section given evidence that despite gains in general awareness, there are still gaps in information that may be key barriers to PrEP uptake and persistence, especially among some of those who might benefit most from PrEP (eg, racial and ethnic minority GBM) [79,80]. Similarly, PrEP-related stigma, or a belief that PrEP users are promiscuous or tainted, may also be a key barrier to PrEP uptake and persistence, particularly among racial and ethnic minority GBM [81,82]. Providing information from a credible source is one technique that can effectively increase knowledge, and specifically, offering information to correct misperceptions about the social consequences of PrEP use may be effective in challenging PrEP stigma [83,84]. In consultation with PrEP providers and past research, we generated a list of five common questions that patients or research participants raise as potential barriers to PrEP uptake, adherence, or persistence: (1) missing a single dose negates protection, (2) side effects are severe, (3) abandoning PrEP is the only choice if one cannot afford it, (4) HIV treatment medications will not work for those who have taken PrEP, and (5) PrEP is only for promiscuous people. One additional misconception was also included because it has been reported among GBM on PrEP who drink alcohol [29]: (6) drinking and taking PrEP may make them sick or reduce PrEP’s efficacy. This section presents each of these misconceptions on an accordion that, when clicked, expands to provide more information and links to helpful sites (eg, links to the US Department of Health and Human Services page for the *Ready, Set, PrEP* program for those who responded consistent with concerns about paying for PrEP). These links are also presented on Game Plan’s final page so that users can reference them later. Footnotes for all information in this section are also included to convey its credibility. This information is presented to both PrEP users and nonusers, given that it may be helpful in addressing barriers to both uptake and adherence or persistence alike.

### Your Drinking Habits

**Figure 3:**
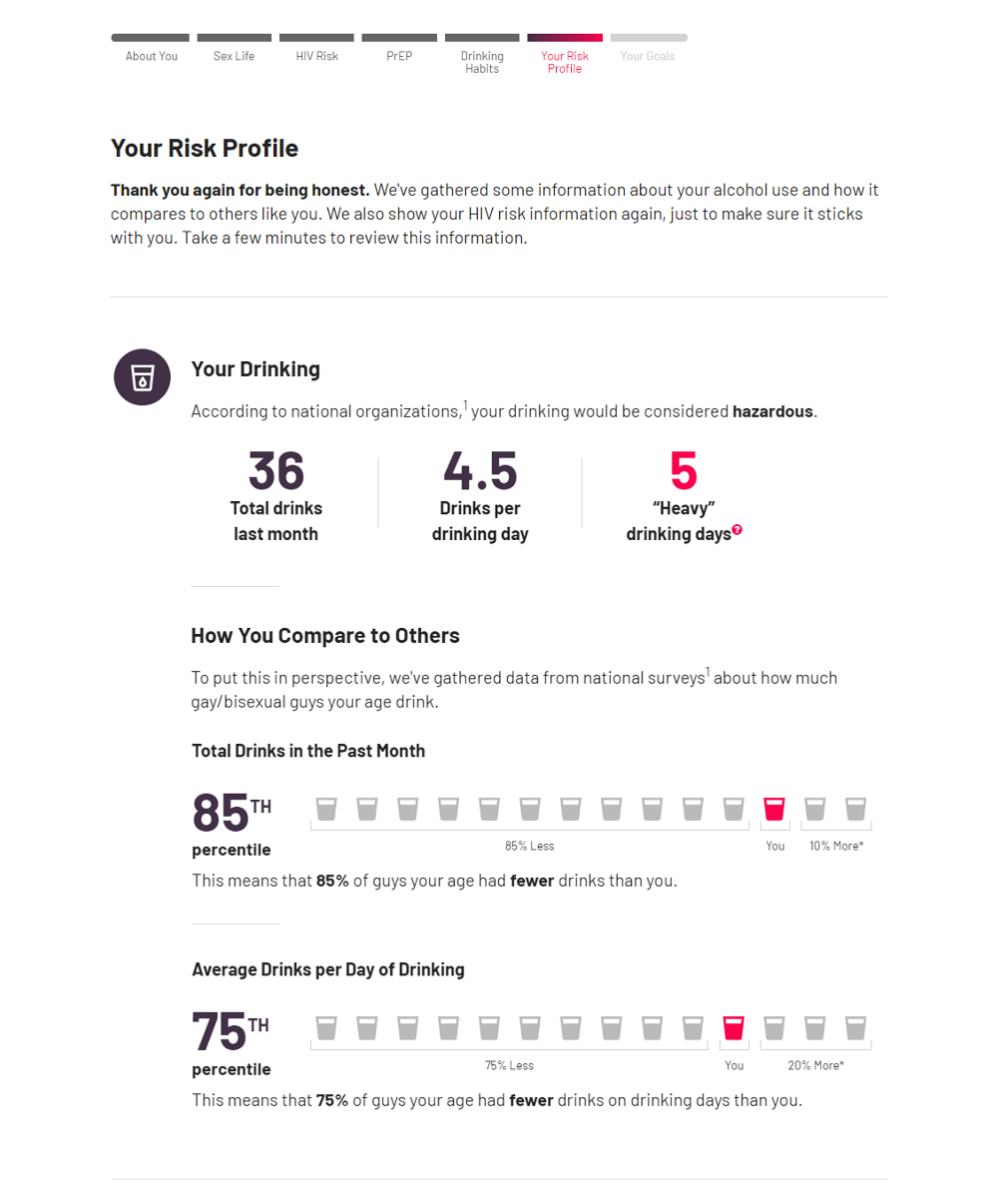
Game Plan—alcohol feedback and social norms. PrEP: pre-exposure prophylaxis.

After sections on sexual behavior, risk, and PrEP, a splash page transitions users to focus on alcohol use. This first section collects information about the users’ recent drinking pattern (past 30 days) and provides feedback that is similar to many other existing personalized feedback interventions for alcohol use [85,86]. The content of this section is also largely unchanged compared with that of the initial version of Game Plan [52]. Briefly, users report their drinking frequency and quantity over the past 30 days using a graduated-frequency approach [87]. To prompt reflection on some common negative consequences of drinking, they are also asked to report whether they have regretted their drinking or experienced an alcohol-related *blackout* within the last month (Figure 3).

The next screens in this section provide information about the link between alcohol use and sexual risk behavior, including the specific ways alcohol plays a role in HIV transmission [26,88], in much the same way as the initial version of Game Plan. It also asks users to identify specific reasons for why they often drink before or during sex [89] and provides specific information challenging beliefs about alcohol’s effects on sexual or romantic behaviors that may produce those motivations. For those using PrEP, these screens also present conditional content that provides information about the ways in which alcohol might interfere with taking PrEP consistently, such as (1) disrupting their normal routines (eg, going to bed or waking up later than usual), (2) causing hangovers that make them feel too sick or indifferent to take their meds, or (3) believing that taking PrEP with alcohol contributed to feeling sick or having worse hangovers. As in previous sections, references are also shown where necessary to ensure that the information provided is perceived as credible.

At the end of this section, users revisit their earlier risk profile with feedback about their drinking included. Users’ overall level of drinking is first classified as *moderate* or *hazardous* according to the National Institute on Alcohol Abuse and Alcoholism guidelines [90]. This screen also provides basic summary feedback about the total number of drinks they consumed, the average number of drinks per drinking day, and the total number of *heavy* drinking days (>5 drinks) they reported in the last month. These data are then compared with those of other GBM in their age group in the United States, highlighting a percentile of GBM that the user drank more than. The goal of this feedback and normative comparison is to again increase intrinsic motivation to change alcohol use by both correcting misperceptions that the user’s current alcohol use level is normative and by highlighting the potential discrepancy between their actual behavior (heavy drinking) and ideal behavior (normative drinking) [91,92]. Feedback about HIV or STI risk from the earlier profile is then presented again to ensure that this information is retained.

### Pros and Cons Exercise

**Figure 4:**
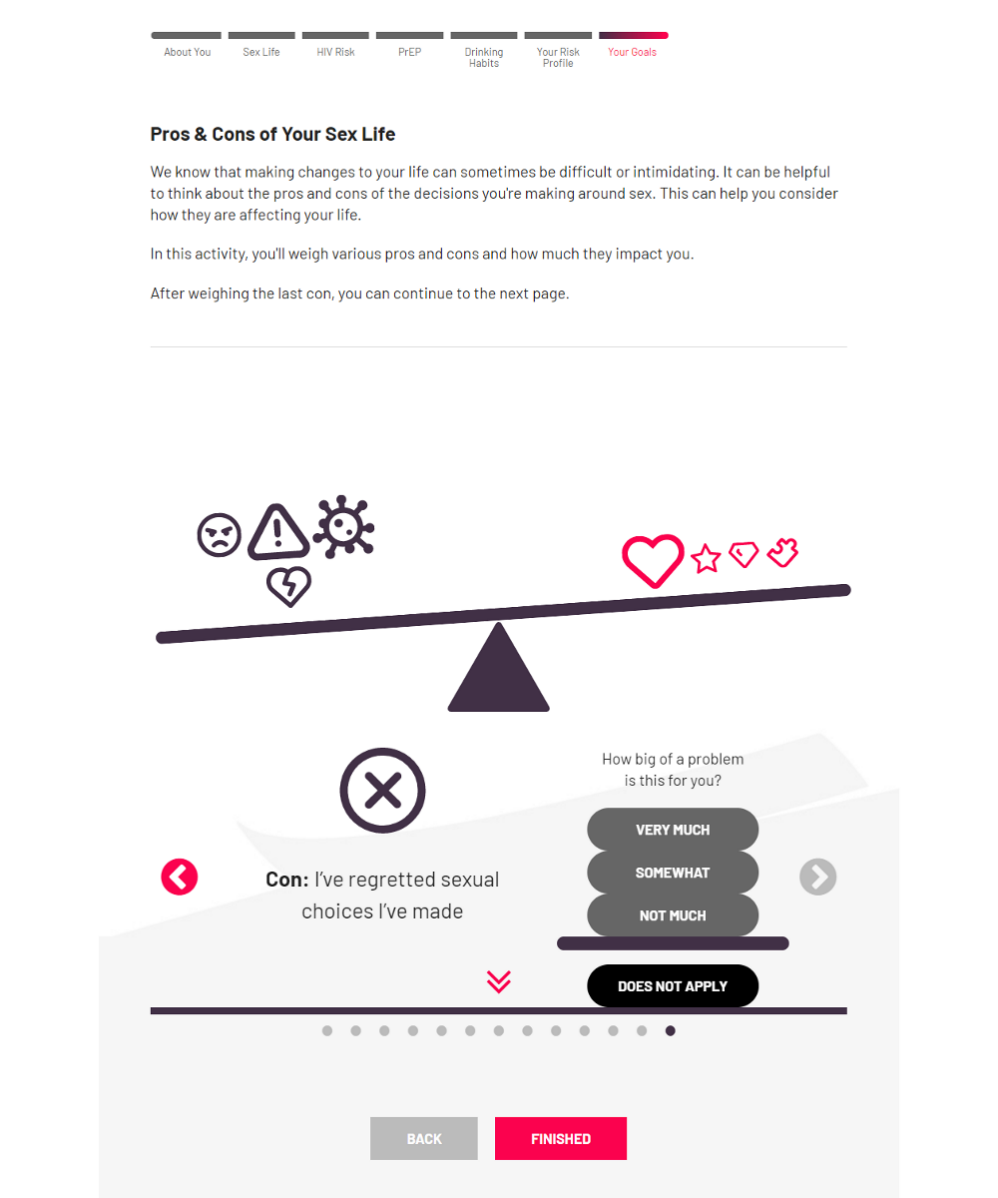
Game Plan—pros and cons exercise. PrEP: pre-exposure prophylaxis.

Users’ motivation to change their choices about sex or alcohol use is then reassessed in a similar way as earlier in the flow. Users who report they are either *not ready* or *not sure* are then directed to a *pros* and *cons* or *decisional balance* exercise [93] that is similar to that in the original Game Plan site. In this exercise, users are presented with a graphical weighing *scale* and asked to scroll through a carousel of *Things I like about my sex life now*. Users indicate whether each pro presented is relevant to them, and if so, its importance to them. Example pros include *My sex life helps me feel connected to my partner* and *My sex life helps me have a better day/night*. Selecting a given pro adds an icon to the scale, and users’ ratings of the importance of each factor determines how much weight the pro adds to the scale. After completing the pros, the users scroll through possible cons. Example cons include *My sex life gets me in trouble with my partner* and *I worry a lot about my choices about sex*. After all possible pros and cons have been selected and rated, a final screen provides users with a summary of the most important pros and cons they chose and feedback text that is conditional based on the direction that their scale was weighted. If the scale is weighted in the direction of cons, the feedback reflects that there seem to be many drawbacks regarding their recent choices about sex. For scales noting at least one con, feedback notes that there are at least some drawbacks that the user may be able to avoid by considering some changes (Figure 4).

### Your Game Plan

**Figure 5:**
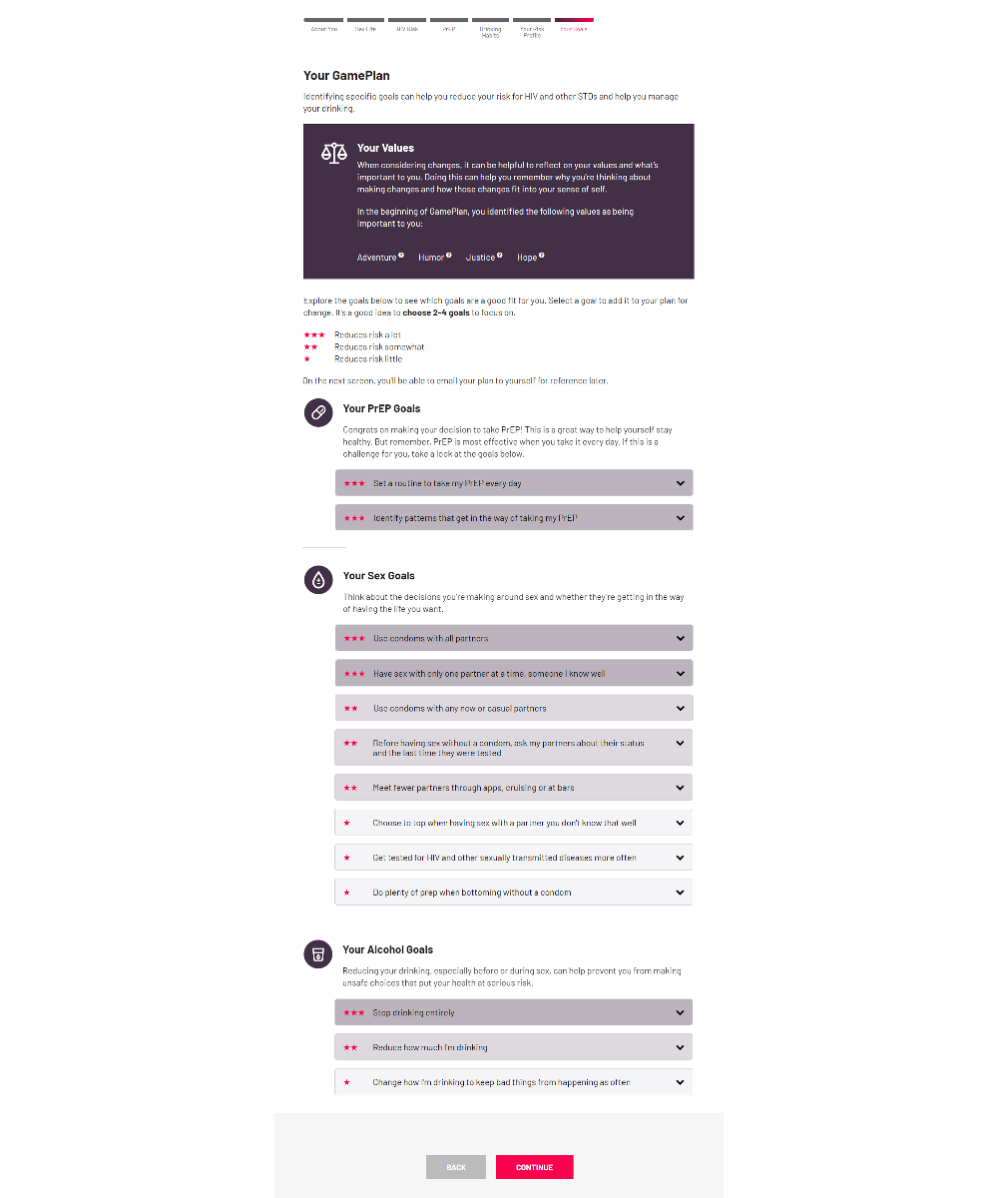
Game Plan—goal setting. PrEP: pre-exposure prophylaxis; STD: sexually transmitted disease.

For users who indicate some level of interest in or ambivalence about change, this section presents a menu of options they can select to help them reduce their sexual risk and alcohol use. In addition to these goals, PrEP users can also select goals to help them take their PrEP more consistently. The section starts by reminding users of the values they selected in the About You section and suggests that keeping them in mind as they consider the possibility of change might help them choose goals that fit them best. All goals shown in this section are prioritized, with those that reduce risk the most presented at the top of each section (Figure 5). Each goal is also assigned a star rating and filled in grayscale, with those that reduce risk considerably given 3 stars and darker gray fill and those that only reduce risk slightly given 1 star and light gray fill. We chose stars and grayscale over other possible color schemes (eg, green, yellow, and orange) to maximize accessibility (eg, for those with colorblindness). Selecting a goal expands an accordion that then asks users to select the most important reasons why they want to make this change and to identify specific steps they will take to achieve this goal. For example, those taking PrEP who choose the goal *Set a routine to take my PrEP every day* can select specific steps such as *Keeping my meds in a prominent place I go every day*, *Set up an alarm or calendar reminder in my phone*, or *Download a medication reminder app*, with links to 2 evidence-based reminder apps provided. For some goals, the available steps are tailored based on the barriers identified by the users. Non-PrEP users are shown goals in two categories: *Your sex goals* and *Your alcohol goals*, with *Take a medication (PrEP) to reduce my risk of HIV* highlighted as a top step. Once users have selected their desired goals (up to 4), motivations, and steps, they can move on to the next screen. This screen congratulates them on their completion of the Game Plan site and affirms their reflection on their sexual health and allows the users to email the plan they made to themselves and to sign up for weekly text messages to check up on the goals they set. Although users can access local resources from anywhere in the site via a navigation pane (eg, for HIV or STI testing, PrEP, medical care, mental health care, and drug or alcohol treatment), links to a page providing these resources also appear on this page.

This section is similar to the change planning steps in MI [63] and involves eliciting users’ potential goals, helping them identify specific steps that can help achieve those goals, and eliciting commitment to that plan [63,94]. Presenting users with several options and encouraging them to choose any that are right for them may also help convey a sense of respect for users’ autonomy. Similarly, presenting several practical ideas for ways to achieve each goal supports users’ self-efficacy, a factor that past studies have shown is linked to PrEP uptake, consistent use, and condom use [25,95]. Finally, encouraging users to select specific goals may also help produce a sense of commitment that builds motivation for change to help users enact their planned changes (Figure 5).

### SMS Text Messaging

On the final page of the Game Plan site, users can enter their phone number to sign up for weekly SMS messages that check in on the goals they set on the site. If they do, the program involves one interaction per week for 12 weeks. A weekly interval was chosen given that most of the behaviors of interest (PrEP adherence, sex, and alcohol use) are likely to be accurately recalled at weekly intervals [96] and to ensure that follow-up was as minimally burdensome as possible for users. Each week, a 2-way interaction was initiated on Monday. The goal of this program is to increase or maintain users’ motivation to start or adhere to PrEP and reduce their sexual risk and alcohol use by providing them with ongoing feedback about their progress toward the goals they set in Game Plan and how their engagement in these behaviors over time affects their risk. For users on PrEP, the program asks participants how many days in the last week they took their PrEP (0 to 7 days) and then either praises them if they took ≥5 doses or offers encouragement if they took less. If users chose goals to increase their PrEP adherence in the Game Plan site, text messages also provide feedback about how well past week adherence aligned with the goals they set, offering praise if it did or encouragement if it did not. For those not on PrEP who set a goal to start PrEP in Game Plan, the program asks whether they started taking PrEP in the last week or not and offers praise if they did and encouragement with a link to the HIV.gov PrEP locator website if they did not.

Next, the program asks a similar set of questions as those in the main site about past week sexual behavior (eg, number of anal sex partners and number of times they had anal sex as bottom and top with and without condoms). The program then provides feedback about how their cumulative *rate* of CAS events reported over each week after completing Game Plan compares with the rate they reported in the year before completing Game Plan. For example, if users report having CAS with an unknown-status partner once each week in the first 2 weeks of the SMS text messaging program and 24 total such events in the past year in the Game Plan, the SMS text messaging program provides feedback that this represents a 117% increase over the previous year. It also provides feedback to users about what this rate would suggest their risk for HIV (if they are not on PrEP) or STIs (if they are on PrEP) might be over the course of a year and how that compares to the rate they were shown on the Game Plan site. If users set goals in Game Plan to reduce their number of sex partners or use condoms with all or status-unknown partners, the SMS text messaging program also gives them feedback about what their *rate* of new partners or CAS events would suggest about their progress toward those goals, praising them if they are reducing these, and encouraging them if they have remained the same or increased.

Finally, the SMS text messaging program asks participants how many standard drinks they consumed over the past week and how many days they drank ≥5 drinks in a single day. It then gives them feedback on how this level of drinking compares with their level over the 30 days before they completed Game Plan. If users had set a goal to reduce their drinking in Game Plan (either reducing how much they were drinking or quitting alcohol entirely), it also gives them feedback about whether their weekly drinking since completing Game Plan represents progress toward those goals or not, praising them if it does and encouraging them if it does not. The SMS text messaging program provides links to local resources in a link at the end of each interaction.

This interaction is similar to several other previous SMS text message–based interventions developed to address each of these outcomes. For example, Liu et al [97] showed that daily SMS text message–based *check-ins* and reminders to take PrEP increased more than twice the participants’ PrEP adherence and follow-up visit attendance. These findings are consistent with a large body of work showing that SMS text message–based interventions also reduce nonadherence to HIV treatment medications and follow-up visits [98]. Fewer such programs have been developed for sexual risk reduction, and evidence of efficacy is mixed [99,100]. However, similar programs developed to help recipients reduce alcohol use show promising effects [101], although the rigor of studies testing stand-alone SMS text messaging interventions for alcohol has been low to date.

## Discussion

### Principal Findings

Deciding to start and remain on PrEP is a complicated decision that is influenced by several structural, interpersonal, and individual factors. Similarly, for those already using PrEP, taking it every day for as long as one is at risk also depends on a variety of factors, such as the ability to pay for it, logistical gaps in renewing and receiving prescriptions, and the capacity to find and set a routine [34]. A web application is not well-suited for addressing all of these barriers. However, individual-level determinants, such as underestimated risk, low PrEP knowledge, high PrEP stigma, and low self-efficacy, also play important roles in PrEP uptake and use [24,25], and as access to PrEP improves and the cost barrier is reduced, these factors may become even more important in optimizing these outcomes. A growing body of research has clearly shown that web-based interventions can change factors such as these and result in meaningful improvements in health outcomes [102,103]. In this study, we created several components of a web application to address these individual-level factors, with the goal of ultimately increasing PrEP uptake among those not using PrEP and improving PrEP adherence and persistence and reducing STIs among those using PrEP. We designed these components and redesigned the broader Game Plan web application with the goal of creating a simple tool that uses evidence- and theory-based strategies that help some GBM who are ambivalent about PrEP to see its utility and use it. We also designed this tool to be brief and feasible for real-world use so that it aligns with how most people access and use health information on the internet [54,55] and could realistically be implemented in the real world, if it is shown to improve outcomes. We also spent considerable effort ensuring that the site was attractive so that users might *want* to use it, a necessary condition for success with digital interventions in the real world.

To date, few internet-facilitated interventions addressing PrEP outcomes exist and among those that do [104], to our knowledge, all are still in the process of being rigorously tested in large efficacy trials. As such, determining how much internet-facilitated interventions might assist other efforts in improving PrEP uptake and use among GBM in the United States is not yet clear. Many similar self-guided, internet-facilitated interventions for alcohol use have been tested, and meta-analyses have shown that they are effective in reducing drinking [105,106]. Most studies have specifically tested these interventions in samples that include those at high risk for alcohol use disorder [107,108], and although this level of care may intuitively seem insufficient for those with such severe problems, the improvement these interventions initiated was not reduced in samples with a higher percentage of participants with more severe alcohol problems. As such, although interventions like Game Plan alone are unlikely to be sufficient to consistently encourage extensive and durable long-term change, particularly in those with severe alcohol use disorders, there is reason to expect that it may be helpful for some at highest risk and could play a role in initiating decisions to seek further help.

### Future Directions

Research on these new components of Game Plan is currently in its preliminary phases. Initial design research with GBM suggests that Game Plan is generally engaging and that users believe they would use it if they encounter it in the real world [56]. However, little is known about the effects of these components on PrEP outcomes. We are currently conducting a pilot randomized controlled trial with 50 heavy drinking GBM who currently use PrEP and have taken <80% (24/30 days) of their prescribed doses in the past month. Participants will be randomly assigned 1:1 to either use Game Plan or an attention-matched control (a general health site that discusses diet and sleep) and followed for 6 months. During the 6-month period, participants will complete web-based surveys assessing PrEP use, sexual behavior, alcohol use, and important antecedents of change for each of these outcomes at baseline and 1, 3, and 6 months. They will also provide dried blood spots collected using kits sent to them in the mail for analysis of tenofovir-diphosphate—a biomarker of PrEP adherence—and phosphatidylethanol—a biomarker of recent alcohol use—at baseline and 3 and 6 months. Finally, we will also collect data on STI diagnoses from the participants’ medical records throughout the study. The primary outcomes will be (1) PrEP adherence or persistence, (2) STI rates, and (3) alcohol use. If the results of this pilot are promising, we plan to pursue support for a full-scale efficacy trial testing of Game Plan’s effects on similar outcomes among GBM in *real-world* PrEP clinics in areas with high HIV incidence. We also recently began a fully powered efficacy trial of Game Plan’s effects on PrEP uptake (among other outcomes) in GBM who are testing for HIV and other STIs using kits delivered to them in the mail. This study will recruit 360 GBM from several high HIV incidence areas in the United States (eg, Atlanta, Miami, and Baltimore) from several web-based platforms (eg, social media and gay-oriented dating apps) and randomize them 1:1 to either use Game Plan or standard of care (access to a 24-hour helpline) when they complete their first test kit. Participants will then be followed for a year, completing quarterly web-based surveys and HIV or STI test kits at 6 and 12 months. These studies will be critical for determining whether a tool like Game Plan can help make a plan to start and take PrEP consistently.

We initially designed Game Plan to provide content to help users reflect and *make* a plan about PrEP because relatively few tools have been explicitly designed so far to boost users’ motivation to reduce their HIV-risk behavior. Therefore, Game Plan currently contains few tools to help users follow through on or maintain those plans. However, users who set a goal to take their PrEP regularly while completing their change plans are shown links to several smartphone apps that were developed to help encourage consistent adherence to medications (eg, Mango Health [TrialCard, Inc] and MediSafe). In the near future, we hope to develop additional features to help users maintain their adherence over time that are unique to PrEP.

### Limitations

Although Game Plan has many strengths, several limitations are important to note. First, Game Plan was primarily developed through user-centered design research conducted with GBM. Risk and social norms feedback are also provided in comparison with other GBM. As such, Game Plan is not appropriate for use with other high-priority populations, including transgender individuals. Second, Game Plan was designed primarily to help those for whom individual-level factors are the primary barriers to successfully using PrEP. Although some content provides simple ways for some users who may have difficulty starting or taking PrEP because of financial or structural barriers to overcome them (eg, by learning about the *Ready, Set, PrEP* program and reaching out themselves), Game Plan’s content is likely to be insufficient for helping these individuals durably address these barriers. Therefore, Game Plan was not intended to replace other critical services (eg, PrEP navigation and provider panel management) and may be best viewed as a tool that could add to these services or provide at least some support when offering these other services is not possible. Finally, one of our highest priorities in developing Game Plan was to create a tool that users might actually use in the *real world*, which could be easily accessed and used in a variety of settings (eg, at home and in clinics). As such, it was essential that Game Plan deliver its techniques within a timeframe that matched how users typically engage with similar tools in their normal lives: a single, brief interaction rather than multiple sessions over time. Given this timeframe, its support for behavior change over time is limited. Although the weekly text messaging feature may add some support for users after they complete their Game Plan, this degree of support is unlikely to be sufficient for many users with more severe or complicated barriers (eg, substance use disorders).

### Summary and Conclusions

In summary, the redesign of the Game Plan site and addition of new components addressing PrEP uptake and use could help encourage some GBM who are not on PrEP to start it or encourage those who already use PrEP to take it more consistently. By showing users how much PrEP could reduce their risk for HIV with consistent use, challenging common myths, eliciting commitment to starting PrEP or using PrEP consistently, and providing practical steps that users can take to accomplish those goals, Game Plan could be a scalable and far-reaching tool that helps some GBM be successful on PrEP. If ongoing research demonstrates its benefit for PrEP outcomes, Game Plan could be a useful option for helping facilitate PrEP-related change in settings where providing evidence-based, face-to-face interventions is difficult.

## Abbreviations

CAS: condomless anal sex
GBM: gay, bisexual, and other men who have sex with men
MI: motivational interviewing
PrEP: pre-exposure prophylaxis
STI: sexually transmitted infection
